# Reassessing the role of high dose cytarabine and mitoxantrone in relapsed/refractory acute myeloid leukemia

**DOI:** 10.18632/oncotarget.27618

**Published:** 2020-06-09

**Authors:** Jonathan Canaani, Meital Nagar, Gabriel Heering, Chen Gefen, Ronit Yerushalmi, Noga Shem-Tov, Yulia Volchek, Drorit Merkel, Abraham Avigdor, Avichai Shimoni, Ninette Amariglio, Gidi Rechavi, Arnon Nagler

**Affiliations:** ^1^ Hematology Division, Chaim Sheba Medical Center, Tel Aviv University, Tel Aviv, Israel

**Keywords:** acute myeloid leukemia, FLT3-ITD, next-generation sequencing

## Abstract

A substantial segment of patients with acute myeloid leukemia (AML) will relapse following an initial response to induction therapy or will prove to be primary refractory. High-dose cytarabine and mitoxantrone (HiDAC/MITO) is an established salvage therapy for these patients. We studied all adult patients with relapsed/refractory (R/R) AML who were treated with HiDAC/MITO in our center between the years 2008-2017. To determine whether responding patients harbored a unique molecular signature, we performed targeted next-generation sequencing (NGS) on a subset of patients. The study cohort consisted of 172 patients with a median age of 54 years (range 18–77). The composite complete remission rate was 58%; 11 patients (6%) died during salvage therapy. Median survival was 11.4 months with a 1-year survival rate of 48%. In multivariate analysis favorable risk cytogenetics [Odds ratio (OR)=0.34, confidence interval (CI) 95%, 0.17–0.68; *P* = 0.002], and de-novo AML (OR = 0.4, CI 95%, 0.16–0.98; *P* = 0.047) were independently associated with a favorable response. Patients who attained a complete remission had a median survival of 43.7 months compared with 5.2 months for refractory patients (*p* < 0.0001). Neither the *FLT3-ITD* and *NPM1* mutational status nor the indication for salvage therapy significantly impacted on the response to HiDAC/MITO salvage. NGS analysis identified 20 different mutations across the myeloid gene spectrum with a distinct *TP53* signature detected in non-responding patients. HiDAC/MITO is an effective salvage regimen in R/R AML, however patients with adverse cytogenetics or secondary disease may not benefit as much from this approach.

## INTRODUCTION

While most patients with acute myeloid leukemia (AML) will achieve an initial remission following induction chemotherapy [1], up to 40% of patients will experience induction failure [2–5], and nearly half of the younger patient population (60 years or younger) achieving a first complete remission (CR1) will eventually relapse with older patients experiencing relapse rates reaching upwards to 80% [6, 7]. Prognosis for this clinically challenging patient segment is quite poor with long term survival being realized in only a small fraction of patients treated both with intensive and non-intensive approaches [8–10]. Notwithstanding the recent introduction of novel agents targeting clinically actionable mutations such as *FLT3-ITD* [11], *IDH1* [12], and *IDH2* [13], many patients with relapsed/refractory (R/R) disease are still currently treated with cytotoxic chemotherapy based salvage regimens. However, at present there is no clearly established standard of care with regard to a specific salvage regimen in patients with R/R AML, indicated by a substantial body of literature published over the last three decades [14–24]. Indeed, in the absence of head-to-head comparisons of the multitude of regimens currently used [25], ascertaining the superiority of a given therapeutic approach and predicting which patient subsets are most likely to benefit from a specific salvage regimen is a central challenge. One of the established salvage protocols for R/R AML patients is high dose cytarabine (HiDAC) and mitoxantrone (MITO) as the initial salvage regimen based on favorable experience with this regimen [26–28]. In this study we endeavored to reassess the clinical efficacy of HiDAC/MITO in 172 R/R AML patients treated in our center and determine clinical and lab parameters of potential predictive value of therapeutic efficacy. Moreover, as next generation sequencing (NGS) is gaining increased acceptance as an innovative modality in AML for genomic classification [29], risk stratification [30], and tracking of minimal residual disease [31], we sought to investigate whether NGS profiling can predict for treatment response in our patients treated with HiDAC/MITO.

## RESULTS

### Patients and baseline characteristics

Between January 2008 and April 2017, a hundred and seventy-two patients were treated with HiDAC/MITO salvage for R/R AML. The median age was 54 years with a range of 18-77 years. Patient disposition with regard to treatment allocation during the study period is delineated in Supplementary Figure 1. As outlined in Table 1, a hundred and forty-four (84%) patients had *de-novo* AML, 24 (14%) had a prior diagnosis of the myelodysplastic syndrome (MDS), and 4 (2%) had an antecedent myeloproliferative neoplasm (MPN). Seventeen (10%) patients had favorable risk cytogenetics, 121 (72%) had intermediate risk cytogenetics, and 30 (18%) had high-risk cytogenetics. Fifty-one patients harbored the *FLT3-ITD* mutation whereas 41 (29%) were *NPM1* mutated. All patients received standard single induction with an anthracycline for 3 days concurrent with continuous infusion of cytarabine at 100 mg/m^2^ for 7 days. Ninety-three (54%) patients were treated with HiDAC/MITO salvage for primary refractory disease, 44 (26%) for disease relapse, and 35 (20%) for relapse following allo-SCT. Concurrent DLI was given to 13 patients. Patients received DLI at a median of 3 days after HiDAC/MITO with a median dose of administered cells of 9.6 × 10^7^ CD3/kg (range 0.5–19.6 × 10^7^ CD3 kg). 8/13 patients achieved CR/CRi (62%), no statistically significant difference in terms of response was observed between patients given DLI and those not receiving DLI (*p* = NS). The median survival was 5.8 months for patients administered DLI (range 2.2–78 months), and survival was not significantly different between groups (*p* = 0.38).

**Table 1:** Baseline characteristics of study population

Clinical Parameter	Entire cohort (N = 172)
Year of diagnosis, median (range)	2013 (2006–2016)
Follow up duration in m, median (range)	16 (0.9–116)
Age in y, median (range)	54 (18–77)
Gender, n(%)	
Male	87 (51)
Female	85 (49)
WBC at diagnosis (×10^9^/L), median (range)	14 (0.2–233)
WBC at time of HiDAC/MITO initiation (×10^9^/L, median (range)	3.7 (0.5–118.1)
Platelet at time of HiDAC/MITO initiation, median (range)	89 (4–581)
Creatinine at time of HiDAC/MITO initiation, median (range)	0.82 (0.27–3.39)
Initial remission duration in m, median (range)	8 (0.5–72)
MRC cytogenetic risk category, n(%)	
Favorable	17 (10)
Intermediate	121 (72)
Adverse	30 (18)
Missing	4
AML type, n(%)	
De-novo	144 (84)
MDS	24 (14)
MPN	4 (2)
Extramedullary disease, n(%)	
No	156 (91)
Yes	16 (9)
*FLT3-ITD* status, n(%)	
Wild type	107 (68)
Mutated	51 (32)
Missing	14
*NPM1* status, n(%)	
Wild type	102 (71)
Mutated	41 (29)
Missing	29
Mutational status, n(%)	
*FLT3*^wt^/*NPM1*^wt^	77 (54)
*FLT3*^wt^/*NPM1*^mut^	23 (16)
*FLT3*^mut^/*NPM1*^wt^	25 (17)
*FLT3*^mut^/*NPM1*^mut^	18 (13)
Induction Chemotherapy, n(%)	
Daunorubicin 45 mg/m^2^	27 (18)
Daunorubicin 60 mg/m^2^	107 (71)
Daunorubicin 90 mg/m^2^	5 (3)
Idarubicin 12 mg/m^2^	9 (6)
Other	2 (1)
Missing	22
Disease status, n(%)	
Primary refractory	93 (54)
Relapse	44 (26)
Relapse following stem cell transplantation	35 (20)
DLI combined with salvage chemotherapy, n(%)	
Yes	13 (8)

WBC: white blood cells; MRC: Medical Research Council; *NPM1*: nucleophosmin1; *FLT3-ITD*: FMS-like tyrosine kinase-3 internal tandem duplication; DLI: donor lymphocyte infusion.

Transplant related data are summarized in Supplementary Table 1, most of the transplanted patients (78%) in the analyzed cohort underwent a 10/10 HLA matched transplant either from a matched sibling or a matched unrelated donor, and 7 patients who relapsed post-transplant underwent a second transplant following salvage treatment with HiDAC/MITO.

### Response to salvage therapy

A hundred patients (58%) achieved a composite complete remission and 61 (36%) were refractory to treatment (Table 2). Eleven (6%) patients died during treatment. For patients achieving a composite complete remission and who did not have a prior allo-SCT the median duration of remission following HiDAC/MITO was 83 days (range 61–251 days). For those patients who relapsed following a prior allo-SCT the median duration of remission duration following HiDAC/MITO was 310 days (range 52-1193 days). A univariate analysis (Supplementary Table 2) was performed to assess the effect of clinical parameters on the likelihood of achieving a complete remission (CR). There was no significant association of gender, WBC count at initial diagnosis, presence of extramedullary disease, type of initial induction chemotherapy, and use of DLI with attainment of a complete remission. The indication for salvage chemotherapy (*i. e.* refractory versus relapsed disease), and the presence of either a *FLT-ITD* or an *NPM1* mutation were also not significantly different between patients who achieved a complete remission versus those who did not. Younger patients were more likely to achieve a CRc, the mean age for patients achieving a CRc was 48 years compared to 54 years for non-responding patients (*p* = 0.019). The cytogenetic risk category was significantly associated with rate of CRc, patients with favorable risk cytogenetics had a 100% rate of CRc, patients with intermediate risk cytogenetics were more likely to be in the CRc group (56%) while those with adverse-risk cytogenetics were more likely to be in the non-responding group (53%) (*p* = 0.001). Type of AML was also significantly different between responders and non-responders whereby patients with *de novo* AML were more likely to respond (63% vs. 37%) while patients with antecedent MDS or MPN were less likely to be in the responding group (37% vs. 63% and 25% vs. 75%, respectively; *p* = 0.028). Table 3 summarizes the multivariate analysis demonstrating that cytogenetic risk category to be significantly associated with likelihood of remission (odds ratio (OR) = 0.34, 95% confidence interval (CI) 0.17–0.68, *P* = 0.002). Type of AML was also significantly associated with likelihood of remission (OR = 0.4, 95% CI 0.16–0.98, *P* = 0.047). There was a statistical trend toward a younger age being associated with response to therapy (OR = 0.98, 95% CI 0.95–1.003, *P* = 0.097).

**Table 2:** Clinical outcomes for patient treated with salvage HiDAC/MITO

Clinical Parameter	
Response to salvage therapy, n (%)	
Refractory	61 (36)
CRc	100 (58)
Induction mortality	11 (6)
Patients still alive, n (%)	65 (38)
Remission duration for patients achieving CRc after salvage Tx, median d (range)	
No prior allo-SCT and no subsequent allo-SCT	120 (61–251)
Prior allo-SCT and not bridged to 2nd allo-SCT	321 (52–1162)
Prior allo-SCT and bridged to 2nd allo-SCT	288 (222–1980)

CRc: composite complete remission; allo-SCT: allogeneic stem cell transplantation.

**Table 3:** Multivariate analysis of factors impacting on response to HiDAC/MITO

	OR	95% CI	*P*
Age at diagnosis	0.98	0.95–1.003	0.097
			
MRC cytogenetic risk category	0.34	0.17–0.68	0.002
			
De novo AML	0.4	0.16–0.98	0.047

MRC: Medical Research Council; OR: odds ratio; CI: confidence interval.

### Survival analysis

**Figure 1:**
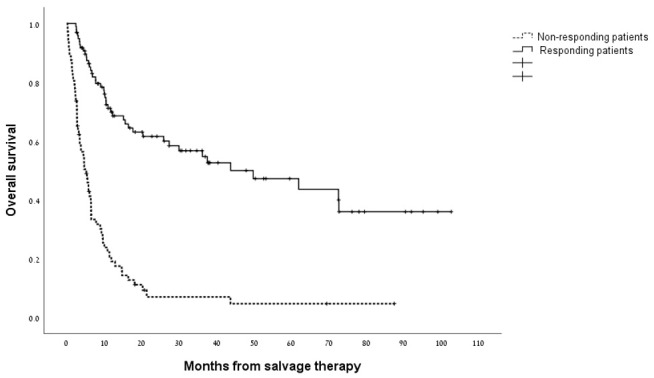
Kaplan–Meier estimate of overall survival in responding patients and non-responding patients to treatment with HiDAC/MITO.

**Figure 2:**
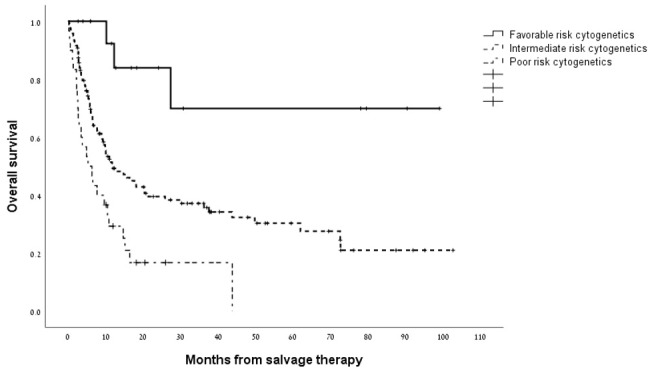
Kaplan–Meier estimate of overall survival according to cytogenetic risk group.

Overall, the median survival for the cohort analyzed as a whole was 11.4 months (95% CI 7.5–15.2 months) with a 1-year survival rate of 48% (Supplementary Figure 2). As shown in Figure 1, the median overall survival for patients who attained a CRc with HiDAC/MITO was 43.7 months (95% CI 14.3-73.2 months) compared with 5.2 months (95% CI 3.5-6.9 months) for patients who were refractory to treatment (*p*<0.0001). The indication for salvage treatment had significant impact on overall survival whereby patients treated with salvage chemotherapy for disease relapse post allo-SCT experienced worse survival compared to patients treated for either relapsed or refractory disease (median survival of 5.8 months versus 25.8 and 14.6 months, respectively; *p* = 0.001). Figure 2 outlines the outcome of patients according to the cytogenetic risk category revealing distinctly worse outcomes for patients with adverse risk cytogenetics compared to their counterparts with intermediate and favorable risk cytogenetic studies (*p* = 0.002). The presence of a *FLT3-ITD* mutation was not significantly associated with overall survival (Supplementary Figure 3). Next, we wanted to assess the duration of remission for patients who responded to HiDAC/MITO. Notably, in patients without a previous allo-SCT, who responded to HiDAC/MITO and did not undergo a subsequent allo-SCT, the median duration of remission was 120 days (range 61–251 days). Conversely, when analyzing the patient subset of post-transplant relapses and who responded to HiDAC/MITO, we observed that patients bridged to a second transplant experienced a significantly longer remission duration (median duration of 288 days; range 222-1980 days, *p* = 0.027) whereas responding patients who were not bridged to a second transplant had a median remission duration of 321 days (range 52-1162 days) with no significant difference between both groups (*p* = 0.18).

### Next generation sequencing

To assess whether the response to HiDAC/MITO could be predicted based on a specific myeloid molecular signature we performed next generation sequencing on a subset of 25 patient samples which were available for sequencing. As shown in Table 4, the NGS studies identified a multitude of mutations in myeloid associated genes. In aggregate, 20 different mutations were detected in both the responding and non-responding groups. Overall, in the responding group, *DNMT3A* (4), *TP53* (2), *KIT* (2), *RUNX1* (2), *IDH2* (2), *KRAS* (2), and *KDMT6A* (2) were the most common identified mutations. In the non-responding group mutations in *TP53* (4), *DNMT3A* (3), and *BCOR* (2) were the most common mutations detected. In three patient samples none of the 54 mutations detectable with the NGS platform used in this study were identified. Non-responding patients were significantly more likely to harbor *TP53* mutations compared to patients who responded to salvage chemotherapy (*p* = 0.026). Of note, five of the six patients with *TP53* mutations harbored adverse risk cytogenetics.

**Table 4:** Mutations detected in relapsed/refractory AML patients grouped by response to HiDAC/MITO

Patient	Clinical response	Mutation (VAF% AA)	Genomic Subgroup
PT1107	Responded to salvage Tx	BCORL1 (100% N1382K)	AML with mutated chromatin or RNA-splicing genes
PT1108	Responsed to salvage Tx	KRAS (11% G12C)NOTCH1 (53% L2457V)	AML with inv(16)(p13.1q22)
PT1125	Responded to salvage Tx	DNMT3A (48% R729W)IDH1 (52% R132C)TP53 (9% P309S)	AML with TP53 mutations and/or chromosomal aneuploidy
PT1130	Responded to salvage Tx	KDM6A (13% E1102K)RUNX1 (8% A123T)	AML with t(8;21)(q22;q22)
PT1132	Responded to salvage Tx	DNMT3A (45% R484fs)NPM1 (45 %W288fs)	AML with *NPM1* mutation
PT1133	Responsed to salvage Tx	DNMT3A (37% R882H)IDH2 (36% R172)ATRX (13% D1681G)	AML with *MLL* fusion genes
PT1138	Responded to salvage Tx	NRAS (96% Q61R)TP53 (8.5% A347D)STAG2 (23% F178l)CBL (46% c.1227+4c>T)	AML with TP53 mutations and/or chromosomal aneuploidy
PT1141	Responsed to salvage Tx	FLT3 (21% V592D)KIT (9% N822K)	AML with t(8;21)(q22;q22)
PT1142	Responded to salvage Tx	KIT (49% Y578C)	AML with inv(16)(p13.1q22)
PT1143	Responsed to salvage Tx	IDH2(47% R172K) KRAS(39% G12D)TET2 (55% P1723S, 48% Y867H)	AML with IDH2^R172^ mutations
PT1126	Responded to salvage Tx	RUNX1 (53% R162K)DNMT3A (39% c.2174-2A>G)SRSF2 (31% R94dupGCC)	AML with mutated chromatin or RNA-splicing genes
PT1149	Responsed to salvage Tx	KDM6A (49% L915S)	AML with no detected driver mutations
PT1153	Responded to salvage Tx	BCOR (24% P624S) WT (13% P390T)	AML with inv(16)(p13.1q22)
P1109	Refractory to salvage Tx	IDH2 (40% R172K)DNMT3A (32% F372indel)KRAS (100% A11T)	AML with TP53 mutations and/or chromosomal aneuploidy
PT1137	Refractory to salvage Tx	DNMT3A (36% L737F)	AML with driver mutations but no detected class-defining lesions
PT1139	Refractory to salvage Tx	DNMT3A (44% R882H)	AML with driver mutations but no detected class-defining lesions
PT1144	Refractory to salvage Tx	NPM1 (15% C.759-1G>C)CSF3R (51% E808K)	AML with NPM1 mutation
PT1145	Refractory to salvage Tx	ATRX (25% Y203*, 16% D207V)CBL (49% c.1227+4C>T)	AML with TP53 mutations and/or chromosomal aneuploidy
PT1147	Refractory to salvage Tx	TP53 (86% Splice Site)BCOR (30% R1532S, 15% H1542D)EZH2 (13% Q420*)KIT (12% I542F)	AML with TP53 mutations and/or chromosomal aneuploidy
PT1151	Refractory to salvage Tx	TP53 (47% R110H)FLT3 (45% D835E)RUNX1 (44% R201*)ETV6 (36% R369W)IKZF1 (40% K58fs*5)TET2 (33% R1452*)	AML with TP53 mutations and/or chromosomal aneuploidy
PT1152	Refractory to salvage Tx	TP53 (18% K114R)KIT (52% V50L)BCOR (8% D1352N)	AML with TP53 mutations and/or chromosomal aneuploidy
PT1128	Refractory to salvage Tx	TP53 (59% T125M)	AML with TP53 mutations and/or chromosomal aneuploidy

VAF: variant allele frequency; AA: amino acid.

## DISCUSSION

Whereas targeting of molecular aberrations and epigenetic dysregulation is an area of flux in AML, standard cytotoxic chemotherapy remains the benchmark for evaluating the efficacy and safety of new therapies in AML. This speaks to the need to reevaluate established cytotoxic based therapies with recent clinical data complemented with the use of novel molecular tools. In this analysis of 172 patients with R/R AML we show that using the HiDAC/MITO regimen in this clinical setting is associated with a high remission rate concomitant to a low treatment related mortality rate. Further, using NGS in a subgroup of patients, our data reveal that our patient population, irrespective of response pattern, is enriched for a broad spectrum of mutations in myeloid malignancy associated genes with a unique *TP53* signature detected in the non-responding patient subset.

R/R AML continues to constitute a formidable challenge in the hemato-oncological arena resulting in generally poor long-term outcomes. The intense clinical efforts in the field are evident by the copious number of clinical studies using both standard and investigational agents. Indeed, only in the past decade several major international collaborations have investigated the roles of vosaroxin [32], elacytarabine [33], laromustine [21], and azacitidine [34] in R/R AML. Despite these and other efforts, response rates and perhaps more importantly survival rates have for the most part shown suboptimal results with commonly used regimens such as MEC [15], FLAG-IDA [35], HiDAC [14], azacitidine [34], and clofarabine [22] combinations achieving responses in the range of 16%-54% and long term survival of less than 12 months for most patients.

These data reaffirm historical publications showing HiDAC/MITO to be a robust and effective salvage regimen for patients with R/R AML, furthermore our current data in the era of modern supportive care and highly effective antimicrobial agents, show superior results to those published earlier. Indeed, these outcomes compare favorably with the Southwest Oncology Group phase III trial showing a 6% therapy related mortality compared with 17%, and a 58% rate compared with 44% [27]. We note that our results concur with historical data published by our center two decades ago showing also CR rates of over 50% [28]. Decidedly, the improvement in outcomes for AML patients as a whole and for R/R patients can be accounted for by the marked improvement in treatment of infections [36, 37] and supportive care [38, 39], also supported by a recent analysis of the European Society for Blood and Marrow Transplantation showing markedly better outcomes for more recently transplanted patients compared to historical results [40]. Analogue studies with R/R AML salvage regimens have identified age at initial diagnosis, duration of CR1, cytogenetic risk group, and no prior allo-SCT as predictors of outcome in this clinical setting [10, 41, 42]. This analysis confirms the validity of these clinical variables also in our patient population with superior remission rates seen in younger patients, those with favorable risk cytogenetics, and in patients with no antecedent myeloid disorder. Notably, we did not find that the presence of *FLT3-ITD* to be predictive of the likelihood of either response or overall survival which diverges from the experience of the PETHEMA group with FLAG-IDA and of the GOELAMS study group which studied outcomes of R/R AML patients with various salvage regimens with combined gemtuzumab ozogamicin [42, 43]. In contrast, a recent analysis from the German-Austrian AML Study Group in over 3,300 R/R patients treated on several prospective salvage regimens also did not identify the presence of a *FLT3-ITD* mutation as predictive of response to salvage therapy although patients harboring this mutation did experience worse overall survival [2]. It is likely the discrepant results regarding the prognostic role of *FLT3-ITD* in R/R AML result from the retrospective nature of these studies as well as the heterogenous composition of patients and salvage regimens studied. Unlike previous publications showing a beneficial impact on response to salvage chemotherapy [44], our results argue that in the setting of R/R AML, mutated *NPM1* status does not contribute to improved outcomes, a supposition supported also by a recent analysis by Schlenk and colleagues [5].

Precision medicine is proving to be a transformative approach in hematology and given the incremental advances realized in the field’s understanding of the genomic landscape in *de novo* AML [29, 45] as well as R/R AML [46], it is of significant importance to molecularly interrogate the clinically challenging population of R/R AML patients. The NGS analysis in 25 patients treated with HiDAC/MITO reveals a wide range of mutations along the myeloid gene spectrum comprising mutations in genes related to chromatin modification, tumor suppression, activated signaling, myeloid transcription factors, and DNA methylation. While our study was not powered sufficiently to detect a statistically significant molecular signature unique to either HiDAC/MITO responsive or non-responsive patients, we did find that the non-responding patient subset was enriched for *TP53* mutations which is in line with previous publications corroborating the detrimental impact of *TP53* in AML patients [47]. Our findings extend on recent studies exploring the pivotal role of NGS studies in investigating resistance mechanisms to novel targeted agents used in AML such as gilteritinib [48], crenolanib [49], and ivosidenib [50]. Notably, these studies and our findings lend further credence to the importance of upfront molecular profiling of patients in order to predict outcome and allow for timely allocation of patients with low chances of responding to a given therapy to clinical trials, perhaps best exemplified by the Beat AML Master Trial [51] randomizing patients to study arms based on baseline mutational data.

Considering the known adverse effect of *TP53* mutations in AML in general, and specifically as our data indicate in R/R AML treated with HiDAC/MITO salvage therapy, it is hoped and anticipated that specific targeting of wild-type and mutated *TP53* via MDM2, the main negative regulator of *TP53,* may improve patient outcomes. Indeed, ongoing clinical studies with MDM2 antagonists such as idasanutlin (NCT02545283), milademetan (NCT03634228), and KRT-232 (NCT03041688) are being actively conducted in R/R AML patients. Furthermore, our data lay further credence to the use of HiDAC/MITO as a chemotherapy backbone in innovative clinical trials incorporating novel agents such as the phase III trial assessing in older patients with R/R AML the combination of HiDAC/MITO and CPI-613, a mitochondrial tricarboxylic acid cycle inhibitor (NCT03504410) which has shown promising activity in a phase 1 study [52] or in combination with the *FLT3* inhibitors quizartinib and crenolanib (NCT03250338 and NCT03250338, respectively).

As this was a retrospective analysis, we acknowledge that inherent biases in data collection may potentially affect interpretation of results and thus merit prudent interpretation. Additionally, it is noted that while in this study refractory disease was defined as failure to achieve remission after one cycle of intensive chemotherapy, there is a plurality of working definitions for refractory disease [4, 6, 53].

In summary, HiDAC/MITO is a highly effective salvage regimen for patients with relapsed/refractory AML and retains an important place in the current armamentarium of therapies for this patient population. Our study emphasizes the significant value of employing NGS modalities for prediction of response to therapy in AML patients.

## MATERIALS AND METHODS

### Study population

This was a retrospective analysis of 172 consecutive patients with R/R AML who were treated with the HiDAC/MITO salvage regimen between January 2008 and April 2017. Adult patients aged ≥ 18 years were eligible for analysis if they had relapsed or refractory disease according to standard international Working Group Criteria [54]. Refractory disease was defined as failure to achieve remission after 1 course of intensive induction chemotherapy. Assessment of cytogenetic risk category was performed according to the modified United Kingdom Medical Research Council criteria [55]. Assignment of patients to specific genomic subgroups was undertaken as previously published [29]. This retrospective analysis was approved by the Institutional Review Board of the Chaim Sheba Medical Center.

### Treatment plan

Patients received intravenous mitoxantrone at 20 mg/m^2^ on days 1-2 over 30 minutes with dose reduction to 15 mg/m^2^ for patients over 60 years of age or those deemed to have clinically significant co-morbidities. Immediately following completion of administration of mitoxantrone, patients received cytarabine at 3 g/m^2^ intravenously over three hours on days 1-5 with dose reduction to 1 g/m^2^ for patients over 60 years of age. Supportive care included antifungal prophylaxis following completion of therapy for the duration of neutropenia.

### Statistical analysis

The primary endpoints of interest in this analysis were the composite complete remission rate (CRc) and survival. Complete remission (CR) was defined as a neutrophil count of ≥ 1000/μL and a platelet count of ≥ 100,000/μL, and less than 5% blasts in the bone marrow, and complete remission with incomplete hematologic recovery (CRi) was defined as all CR criteria except for residual neutropenia (<1.0 × 10^9^/L[1000/μL]) or thrombocytopenia (<100 × 10^9^/L [100,000/μL]) [6]. Induction mortality was defined as any death occurring within 30 days of administration of salvage chemotherapy. Overall survival was calculated from the initial day of salvage therapy to death from any cause or to time of last follow up. Univariate analyses were performed using *T*-Test for continuous variables, and Fisher’s Exact test and Pearson’s chi-squared for assessment of categorical variables. Probability of overall survival was determined using the Kaplan–Meier estimate, and comparison of survival between groups was assessed with the log-rank test. Multivariate analyses using logistic regression were performed using age, patient gender, WBC at diagnosis, initial remission duration, cytogenetic risk category, type of AML (de novo/secondary), presence of extramedullary disease, *FLT3-ITD* and *NPM1* status, type of induction chemotherapy, time from diagnosis to allo-SCT, indication for salvage chemotherapy (refractory, relapsed, post-stem cell transplantation relapse), and use of donor lymphocyte infusion (DLI) with salvage chemotherapy as covariates. All tests were two-sided with the type I error rate fixed at 0.05 for the determination of factors associated with time-to-event outcomes. Statistical analyses were performed using the SPSS 25.0 (SPSS Inc, Chicago, IL, USA).

### Targeted next-generation sequencing

Using bone marrow samples obtained at the time of diagnosis we performed targeted next-generation sequencing (NGS) of 54 myeloid cancer associated genes with the Illumina TruSight Myeloid Sequencing Panel, following the manufacturer’s protocol. Briefly, high quality DNA was extracted using the QIAcube (Qiagen, Venlo, The Netherlands). DNA concentration was measured by Qubit Fluorometric Quantitation (Thermo Fisher Scientific, Wilmington, DE). The NGS libraries were sequenced on an Illumina MiSeq System (Illumina, San Diego, CA). Reads were aligned to the human reference genome version 19 (hg19) to create BAM files. The somatic variant caller was then performed for variant analysis of the specific regions in the manifest. Single nucleotide variants (SNVs) and insertions-deletions (indels) at diagnosis were analyzed using the Illumina MiSeq Reporter software and subsequently with Ingenuity software (Qiagen). Variant allele frequencies (VAF) of mutations were calculated as the ratio between the number of mutant and total reads.

## SUPPLEMENTARY MATERIALS



## Abbreviations

AML: acute myeloid leukemia
CR: complete remission
HiDAC/MITO: high-dose cytarabine and mitoxantrone
NGS: next-generation sequencing
OS: overall survival
CI: confidence interval
R/R: relapsed/refractory
allo-SCT: allogeneic stem cell transplantation
