# Supplementary material for: Reassessing the role of high dose cytarabine and mitoxantrone in relapsed/refractory acute myeloid leukemia
Source: Oncotarget. 2020 Jun 9;11(23):2233–45. doi: 10.18632/oncotarget.27618 (PMC7289527; doi:10.18632/oncotarget.27618)
Supplement: Supplementary file 1 [file oncotarget-11-2233-s001.pdf]

Reassessing the role of high dose cytarabine and mitoxantrone in relapsed/refractory acute myeloid leukemia

SUPPLEMENTARY MATERIALS

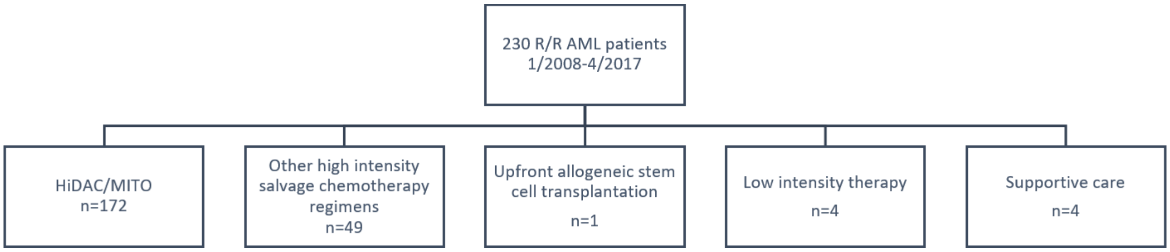

Supplementary Figure 1: Patient disposition.

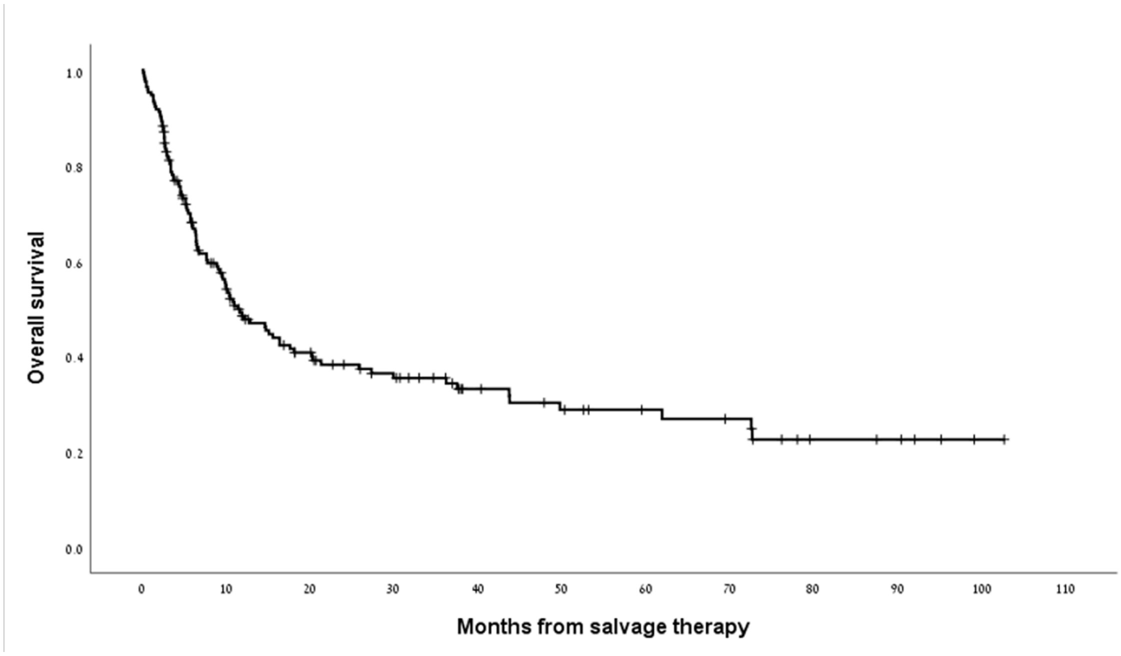

Supplementary Figure 2: Kaplan-Meier estimate of overall survival in 172 patients treated with HiDAC/MITO.

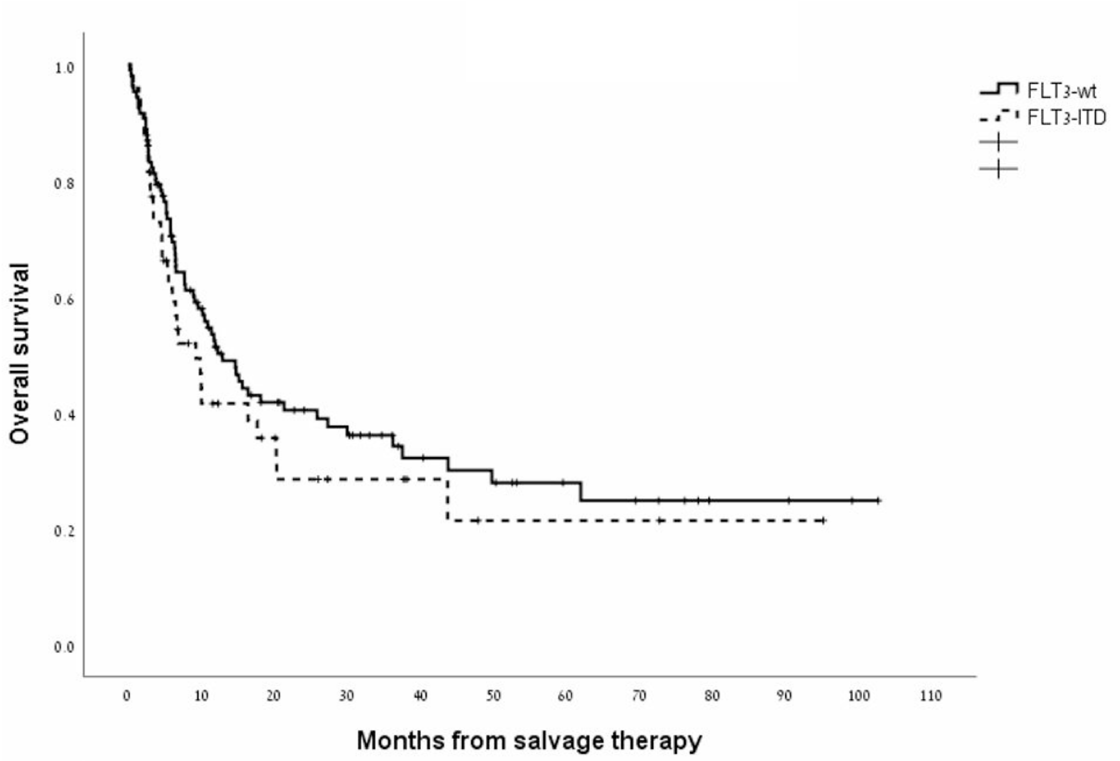

Supplementary Figure 3: Kaplan–Meier estimate of overall survival according to FLT3-ITD mutational status.

Supplementary Table 1: Transplant data

Clinical Parameter	Entire cohort (N = 151)
Year of transplant, median (range)	2013 (2006–2017)
Conditioning regimen, n (%)	
Myeloablative	121 (80)
Reduced intensity	30 (20)
Donor source	
MSD 10/10	63 (42)
MUD 10/10	55 (36)
Haplo	5 (3)
MMURD	24 (16)
Cord blood	4 (3)
Bridging to second Transplant	7

MSD: matched sibling donor; MUD: matched unrelated donor; Haplo: haploidentical donor; MMURD: mismatched unrelated donor.

Supplementary Table 2: Univariate analysis of factors impacting on response to salvage chemotherapy

Clinical Parameter	Responders (N = 100)	Non-responders (N = 72)	*P*
Age in y, mean (std deviation)	48.9 (15.4)	54.3 (14.1)	0.019
Gender, n(%)			0.6
Male	49 (56)	38 (44)	
Female	51 (60)	34 (40)	
WBC at diagnosis, mean (std deviation)	40.0 (53.5)	37.3 (53.2)	0.7
Initial remission duration in m, mean	12.8 (3.8)	6.7 (3.8)	0.033
MRC cytogenetic risk category, n(%)			0.001
Favorable	17 (100)	0	
Intermediate	68 (56)	53 (44)	
Adverse	14 (47)	16 (53)	
Type of AML, n(%)			0.028
De-novo	90 (63)	54 (37)	
MDS	9 (37)	15 (63)	
MPN	1 (25)	3 (75)	
Extramedullary disease, n(%)			*NS*
No	91 (58)	65 (42)	
Yes	9 (56)	7 (44)	
FLT3-ITD status, n(%)			0.16
Wild type	66 (61)	41 (38)	
Mutated	25 (49)	26 (51)	
NPM1 status, n(%)			*NS*
Wild type	58 (57)	44 (43)	
Mutated	24 (59)	17 (41)	
Missing			
Induction Chemotherapy, n (%)			0.17
Daunorubicin 45 mg/m^2	13 (48)	14 (52)	
Daunorubicin 60 mg/m^2	67 (63)	40 (37)	
Daunorubicin 90 mg/m^2	2 (40)	3 (60)	

Idarubicin 12 mg/m^2	3 (33)	6 (67)	
Other	2 (100)	0	
Indication for salvage chemotherapy, n(%)			0.12
Primary refractory	52 (56)	41 (44)	
Relapse	31 (71)	13 (29)	
Relapse following stem cell transplantation	17 (49)	18 (51)	
DLI combined with salvage chemotherapy, n(%)			*NS*
Yes	8 (62)	5 (38)	
No	87 (63)	51 (37)	

WBC: white blood cells; ELN: European LeukemiaNet; NPM1: nucleophosmin1; FLT3-ITD: FMS-like tyrosine kinase-3 internal tandem duplication; CR1: first complete remission; CMV: cytomegalovirus.
